# Editorial: Immunologic Mechanisms of Myeloid Neoplasms

**DOI:** 10.3389/fonc.2022.949633

**Published:** 2022-06-15

**Authors:** Bruno Fattizzo, Matteo Claudio Da Vià, Austin G. Kulasekararaj

**Affiliations:** ^1^ Hematology Unit, Fondazione IRCCS Ca’ Granda Ospedale Maggiore Policlinico, Milan, Italy; ^2^ Department of Oncology and Hemato-Oncology, University of Milan, Milan, Italy; ^3^ Department of Hematology, King’s College Hospital, London, United Kingdom

**Keywords:** myeloid neoplasm, immune system, immunomodulatory, neoplastic hematopoietic stem cell, single cell analysis

Myeloid neoplasms (MN), namely myelodysplastic syndromes (MDS), myeloproliferative neoplasms (MPN), and acute myeloid leukemias (AML) are characterized by disrupted myelopoiesis encompassing increased apoptosis of bone marrow (BM) progenitors, differentiation arrest and increased proliferation (1). This results in either peripheral cytopenia (with fatigue, bleeding, and infectious risk), or in hyperproliferative phenotype (with splenomegaly, high blood counts, and thrombosis). Along with the “first genetic hit” that may happen several years before disease onset in a hematopoietic stem cell (2), the surrounding immunologic niche seems to play a pivotal role in the subsequent disease development. Clinically, the immune system disruption is evidenced by an increased incidence of autoimmune phenomena in MN, which may worsen the degree of cytopenia (particularly anemia and thrombocytopenia) and respond to immunosuppressive therapy (3–5). From a pathogenic point of view, bone marrow hematopoietic stem cells are strongly regulated by the crosstalk with the surrounding microenvironment and its components, including mesenchymal stem cells, lymphocytes, and macrophages (**Figure 1**) (6). Several alterations of these cells have been described in MN, and it is not clear whether they are the cause or consequence of disease development and progression. Furthermore, niche disruption might sustain pancytopenia and promote the accumulation of molecular alterations that lead to leukemic evolution (4, 6, 7). Finally, immunologic alterations might in turn be potential targets for novel biologic drugs (8). In this Research Topic the above-mentioned points have been addressed by eleven articles focusing on pathogenic, prognostic, and therapeutic implications of immune system disruption in MN.

**Figure 1:**
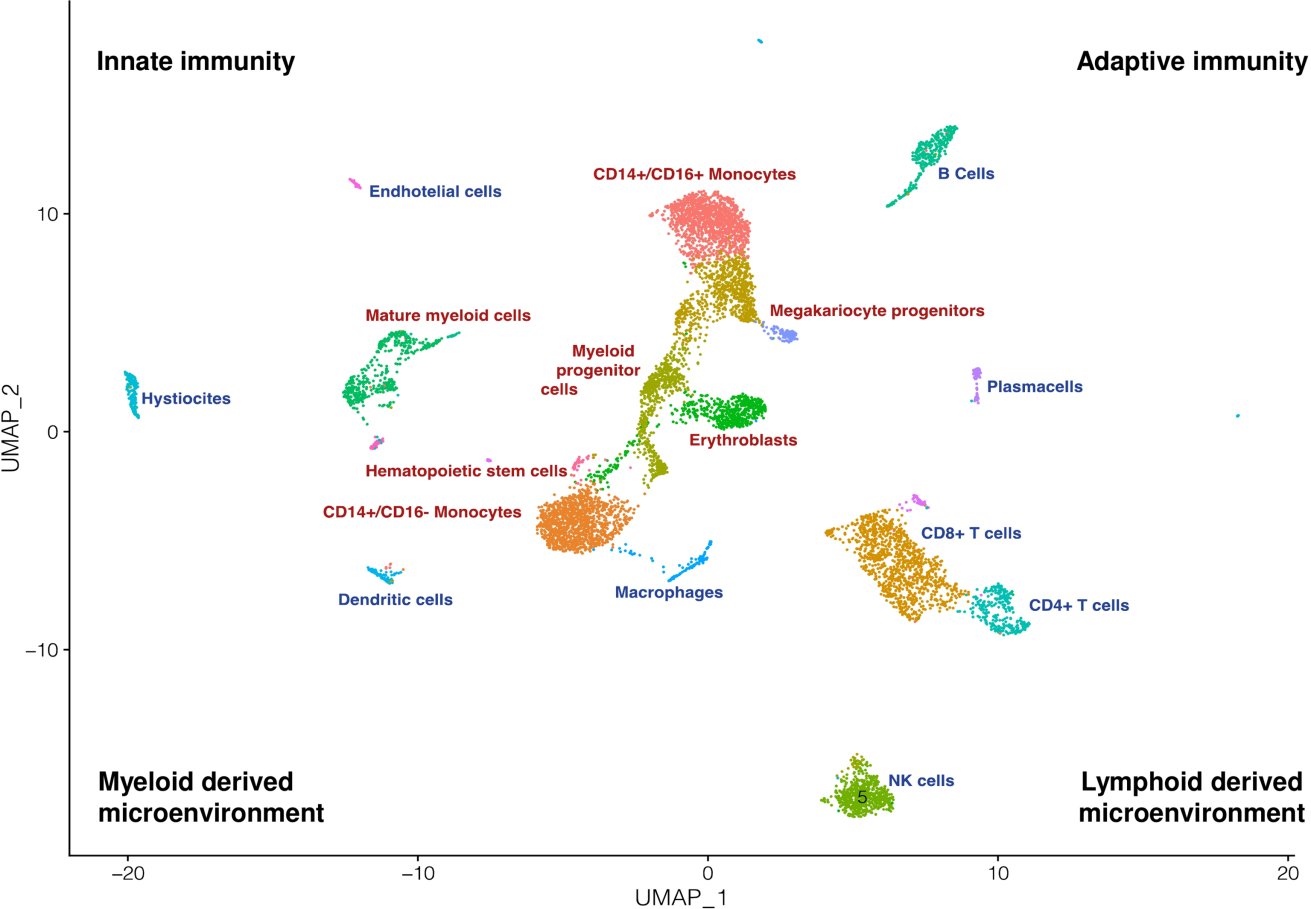
The myeloid compartment and its microenvironment. Bone marrow is a tissue defined by a high cellular and biological complexity. This UMAP recapitulates how myeloid cells (red text) are surrounded and in strict connection with a complex and multifaceted immune microenvironment (blue text).

Barcellini and Fattizzo asked themselves the “egg or chicken” question as to whether immune phenomena comes before or after MN. They examined their epidemiological association, and discussed that autoimmunity and immunodeficiency are the two faces of a dysregulated immune tolerance and surveillance possibly resulting in tumor escape and infections. Alterations of the microbiota and of mesenchymal stem cells in MN are also discussed to highlight the importance of a permissive microenvironment for tumor growth. Finally, the authors highlight how novel therapies for MN (including checkpoint inhibitors and chimeric antigen receptor T-cells) may increase autoimmune phenomena.

Cominal et al., focused on Philadelphia chromosome-negative MPN that display inflammatory alterations of BM niche. They studied BM soluble mediator signatures using a multiplex assay and found a distinctive profile in polycythemia vera with increased levels of chemokines, and growth factors compared to essential thrombocytopenia and primary myelofibrosis. Deregulation of soluble mediators was associated with abnormal blood counts, thrombosis, treatment status and risk stratification and this might represent a therapeutic target. Additionally, JAK inhibitors also affect the levels of inflammatory cytokines in MPN patients, as described by Cattaneo and Iurlo. They also discussed how these drugs affect several components of the innate and adaptive immune systems such as dendritic cells, natural killer cells, T helper cells, and regulatory T cells, resulting in a level of immune deficiency with increased infectious risk.

Sciumè et al., focused on another rare “proliferating” condition: systemic mastocytosis. They described a *KIT D816V* mutated patient who evolved into MN with PDGFRA rearrangement and responded to imatinib therapy; they discuss how immunological mechanisms may play a role in promoting clonal prevalence of one entity (mastocytosis) over the other (MN).

The clinical and prognostic aspects of the concomitant presence of distinct hematological clonal entities was further addressed by Bucelli et al., who described a large series of patients with co-occurrence of myeloid and lymphoid neoplasms. Patients mainly suffered from MPN with associated non-Hodgkin lymphomas; nearly a half required anti-lymphoma therapy and 1/3 experienced a high-grade infection that was significantly associated with mortality.

Whether the myeloid and lymphoid clones share a common origin or develop autonomously is still debated, and another interesting example is the association of large granular lymphocyte (LGL) expansion with MN and BM failure syndromes. Our group performed a literature review and discussed how LGL clones, found in up to 1/3 of MN, are associated with deeper cytopenia (likely through immune mediated apoptosis) and good response to immunosuppression. Far from being innocent bystander, LGL clones may contribute to immunosurveillance, as their depletion after immunosuppression may favor leukemic escape.

Focusing on AML, Li et al., developed and validated an innovative prognostic model based on a novel immune-17 signature derived from transcriptome data from The Cancer Genome Atlas (TCGA) and The Genotype-Tissue Expression (GTEx) databases. They confirmed that immune biology processes and transcriptional dysregulations are critical factors in the development of AML. Interestingly, the incorporation of the immune-17 signature to the ELN2017 risk score improved patient stratification. This immune signature may be therapeutically exploited, as described by Sun Yao et al., that treated an AML patient with PD-1 blockade in combination with azacytidine after allogeneic hematopoietic stem cell transplantation; these strategies that reactivate anti-leukemic immune surveillance may in turn result in devastating autoimmune/autoinflammatory responses, as in the case described who developed fatal graft versus host disease.

Moving to innate immunity effectors, Razanamahery et al., described a case of Erdheim–Chester disease (ECD), a rare histiocytosis, characterized by somatic mutations of MAP-kinase pathway in CD14+ monocytes. They found a correlation between disease activity and increased CD14++CD16− “classical monocyte” and decreased CD14lowCD16++ “non-classical monocyte” highlighting the contribution of a phenotype switch of innate immunity in this rare disease.

Another very rare condition associated with autoimmunity and MN is paroxysmal nocturnal hemoglobinuria (PNH). Giannotta et al., reported a patient with MPN who developed clinically overt PNH requiring anti-complement therapy. They discuss that the selection and expansion of PNH clones in MPN is likely to be ascribed to the same immunological bottlenecks described in BMF: autoimmunity against BM precursors, toxicity of therapies, and acquirement of cooperative somatic mutations.

Finally, Caprioli et al., described how the use of single-cell technologies represent powerful tools to assess the cellular composition of the complex tumour ecosystem and its immune environment (**Figure 1**), to dissect interactions between neoplastic and non-neoplastic components, and to decipher their functional heterogeneity and plasticity. In addition, recent progress in multi-omics approaches provide an unprecedented opportunity to study multiple molecular layers (DNA, RNA, proteins) at the level of single-cell or single cellular clones during disease evolution or in response to therapy. Applying single-cell technologies to MN holds the promise to uncover novel cell subsets or phenotypic states and highlight the connections between clonal evolution and immune escape, which is crucial to fully understand disease progression and therapeutic resistance.

In conclusion, this Research Topic highlights the multifaceted immunologic aspects of pathogenesis, clinical course, and treatment of MN. This expanding field will increasingly benefit from sophisticated molecular tools to further identify druggable pathways/targets and optimize management of MN and other rare entities.

## Author Contributions

All authors listed have made a substantial, direct, and intellectual contribution to the work and approved it for publication.

## Conflict of Interest

The authors declare that the research was conducted in the absence of any commercial or financial relationships that could be construed as a potential conflict of interest.

## Publisher’s Note

All claims expressed in this article are solely those of the authors and do not necessarily represent those of their affiliated organizations, or those of the publisher, the editors and the reviewers. Any product that may be evaluated in this article, or claim that may be made by its manufacturer, is not guaranteed or endorsed by the publisher.
